# Reactive Infectious Mucocutaneous Eruption (RIME) Associated with *Mycoplasma pneumoniae*: Clinical and Immunological Insights from Pediatric Cases

**DOI:** 10.3390/microorganisms14020364

**Published:** 2026-02-04

**Authors:** David M. Matea, Raluca Isac, Estera Boeriu, Patricia Urtila, Gabriela Doros, Mihaela Bataneant, Andrada L. Oprisoni, Smaranda T. Arghirescu

**Affiliations:** 1 Doctoral School, University of Medicine and Pharmacy “Victor Babes”, 300041 Timisoara, Romania; david.matea@umft.ro; 2 Emergency Clinical Hospital for Children “Louis Turcanu”, 300011 Timisoara, Romania; estera.boeriu@umft.ro (E.B.); patricia.urtila@umft.ro (P.U.); doros.gabriela@umft.ro (G.D.); oprisoni.licinia@umft.ro (A.L.O.); arghirescu.smaranda@umft.ro (S.T.A.); 3 Department of Pediatrics, University of Medicine and Pharmacy “Victor Babes”, 300041 Timisoara, Romania

**Keywords:** RIME, MIRM, *Mycoplasma pneumoniae*, pediatric mucositis

## Abstract

Reactive infectious mucocutaneous eruption (RIME) is a rare pediatric condition characterized by severe mucositis, minimal cutaneous involvement, and an infectious rather than drug-induced etiology. *Mycoplasma pneumoniae* (*M. Pneumoniae*) represents the most frequently identified trigger, although an increasing number of alternative pathogens have been reported. Its clinical overlap with Stevens–Johnson syndrome (SJS) makes early recognition difficult. We reviewed literature data on the topic and described our center’s experience with three pediatric cases of *M. pneumoniae*-associated RIME. Medical records, laboratory results, and imaging were systematically analyzed. All patients were male, aged 2 to 12 years and originated from rural communities. Etiologic confirmation was achieved via *M. pneumoniae* IgM serology and/or polymerase chain reaction. Clinical exam modifications included multi-site mucositis (oral, ocular, genital) with variable skin involvement: absent in one case, a solitary palm ulcer in another, and widespread rash in the third. One patient required two hospitalizations within a six-month interval, confirming the possible relapsing phenotype of RIME. Another patient developed pneumonia, sepsis, and systemic inflammation. All received macrolide therapy, antifungals, mucosal supportive care, and systemic management as indicated. Recovery occurred within 10–21 days, with one patient exhibiting skin hyperpigmentation. These cases illustrate the heterogeneity of RIME, emphasize the importance of prompt recognition, etiology confirmation, and multidisciplinary management. RIME is a rare clinical condition in pediatric population, an uncommon but significant mucocutaneous clinical entity, important to be acknowledged by clinicians as a complication and/or extra-pulmonary manifestation of *M. pneumoniae* infection.

## 1. Introduction

*Mycoplasma pneumoniae* (*M. pneumoniae*) is a human pathogen characterized by the absence of a peptidoglycan cell wall, belonging to the class *Mollicutes*, among the smallest organisms yet discovered [1]. The lack of rigid cell walls and peptidoglycan structure is responsible for the organism’s intrinsic resistance to β-lactam antibiotics, while susceptibility to macrolides, tetracyclines, and fluoroquinolones is retained [1,2]. Transmission occurs via respiratory droplets, resulting in both endemic circulation and periodic epidemic outbreaks in school-aged children [3]. Pathogenesis is characterized by and/or penetration of respiratory epithelial cells, followed by cytotoxic injury and immune-mediated inflammation that can extend beyond the lungs and have been implicated in *M. pneumoniae*-related extra-pulmonary diseases (MpEDs) [4,5,6,7].

From a clinical point of view, *M. pneumoniae* infection includes a broad spectrum of respiratory infections, ranging from mild upper respiratory symptoms like sore throat and congestion to more severe lower respiratory infections such as bronchitis and pneumonia (“walking pneumonia”) [3,8,9]. Extra-pulmonary involvement may represent a clinically significant burden, particularly in children and young adults [7,8,9]. In independent studies of hospitalized children with a positive swab sample for *M. pneumoniae*, Gordon et al. and Biagi et al. reported extra-pulmonary manifestations in 88 of 332 patients (26%) and in 74 of 145 patients (51%), respectively; the most common manifestation involved the gastrointestinal system followed by dermatological manifestations [10,11]. Comparable findings were presented by Søndergaard et al. in a retrospective analysis of 134 children with a positive *M. pneumoniae* swab who were treated during the 2010–2011 epidemic in Denmark, with extra-pulmonary symptoms present in 54% of cases [12]. In contrast, the French MYCADO study, conducted by Gavaud et al. and involving 1309 hospitalized adults with positive *M. pneumoniae* swabs, reported extra-pulmonary involvement in only 156 patients (11.9%) [13]. Collectively, these data support the observation that extra-pulmonary manifestations of *M. pneumoniae* infection are more prevalent in pediatric patients than in adults, a disparity that may reflect age-related differences in immune responses and clinical presentation [13]. Other reported extra-pulmonary manifestations of *M. pneumoniae* infection include neurological complications (encephalitis, meningitis, Guillain–Barré syndrome, myelitis), cardiac involvement (myocarditis, pericarditis, arrhythmias), hematologic abnormalities (hemolytic anemia, thrombocytopenia, coagulopathies), renal injury (acute glomerulonephritis, interstitial nephritis), and musculoskeletal disorders (arthritis, myositis) [10,11,13].

With mucocutaneous involvement being an important extra-pulmonary manifestation of *M. pneumoniae* infection, Canavan et al. proposed the designation “Mycoplasma-induced rash and mucositis” (MIRM) as a distinct clinical entity [4]. MIRM is defined by: (1) evidence of *M. pneumoniae* infection (clinical and laboratory), (2) involvement of two or more mucosal sites, and (3) epidermal detachment affecting < 10% of body surface area (BSA). Cutaneous manifestations vary according to the clinical subtype of MIRM, which may be classified as classical, sine rash, or severe forms [4].

More recently, the term reactive infectious mucocutaneous eruption (RIME) has been introduced to encompass a broader spectrum of infectious triggers. In addition to *M. pneumoniae*, RIME has been reported in association with a broad range of infectious triggers, including bacterial pathogens such as *Chlamydia pneumoniae* and group A *Streptococcus*, as well as viral agents including adenovirus, influenza A and B viruses, parainfluenza viruses, human metapneumovirus, enteroviruses, norovirus, Epstein–Barr virus, cytomegalovirus, human herpesvirus 6, and coronaviruses (including NL63 and SARS-CoV-2), supporting its classification as a pathogen-independent, infection-driven mucocutaneous syndrome [4,5,6,9,14,15,16,17,18,19,20,21,22,23,24].

Even if RIME is considered a rare condition, its global prevalence in the pediatric population remains unknown, owing to the scarcity of cases and the predominance of case reports and small case series in the literature [6,9,20,21,25]. In one study of children with community-acquired pneumonia, 22.7% of patients were reported to develop mucocutaneous disease, of whom 6.8% fulfilled the diagnostic criteria for RIME [26]. Recent pediatric literature (2023–2024) has documented a growing recognition of RIME, particularly during *M. pneumoniae* outbreaks, more frequent after the COVID pandemic [6,14,27,28,29,30].

The diagnostic criteria for RIME are similar to MIRM (acute mucositis affecting at least two sites, a recent/concomitant respiratory infection, the absence of recent drug exposure, and limited skin involvement (<10% BSA)) [4,16,31,32,33,34]. While MIRM is now recognized as a distinct clinical entity separate from erythema multiforme (EM) and Stevens–Johnson syndrome (SJS), it is considered a specific subtype within the larger category of RIME [33,34]. Historically, MIRM has been referred to as *M. pneumoniae*-associated SJS, *M. pneumoniae* mucositis, atypical SJS, incomplete SJS, Fuch’s syndrome, recurrent multifocal mucositis, and recurrent SJS-like disease [33,34,35].

MIRM displays minimal epidermal necrosis, a clear infectious etiology, and a more favorable prognosis compared with SJS or toxic epidermal necrolysis (TEN) [18,31,36,37]. Isolating RIME from other mucocutaneous syndromes such as SJS, TEN, and major multiform erythema (EMM) is essential, given their distinct etiologies, prognostic implications, and therapeutic approaches [9,18,31,37,38,39,40]. Both SJS and TEN are classically drug-mediated clinical entities, with extensive epidermal necrosis developing in 1–3 weeks after drug exposure. Antibiotics (sulfamethoxazole-trimethoprim, amoxicillin, ampicillin, cephalosporin, ciprofloxacin, levofloxacin, tetracycline), anticonvulsant/antiepileptic (phenytoin, carbamazepine, phenobarbital, valproic acid), non-steroid anti-inflammatory drugs (NSAIDs) (piroxicam, tenoxicam, naproxen) and allopurinol in the Asian population have been known as agents related to SJS/TEN development [36,38,39,40]. EMM is usually triggered by herpes simplex virus (HSV) and is characterized by target lesions (three zones: central dusky area, pale edematous ring, outer erythematous halo) [40]. The disease is known to display a mucosal predominance and limited epidermal detachment [40].

Significant differences among RIME, SJS, TEN, and EMM are summarized in Table 1, incorporating clinical distribution, extent of epidermal loss and histopathologic patterns. Recognition of these distinguishing features prevents misdiagnosis and avoids unnecessary withdrawal or avoidance of medications [31,36,37,38,39,40].

In RIME, mucositis typically ranges between Grade 2 and Grade 3, according to the World Health Organization (WHO) classification with multi-site involvement (oral, ocular, genital) [27,28,30,41,44,45,46]. The severity of mucosal injury has been shown to correlate directly with disease burden, duration of hospitalization, and the requirement for multidisciplinary management [45,46]. Oral mucositis is most commonly associated with cancer treatment, including chemotherapy and head and neck radiotherapy, particularly as a consequence to drugs affecting DNA synthesis. In immunocompetent hosts, oral mucositis can be caused by infectious agents, including: viruses (HSV, Coxsackievirus, varicella-zoster virus, cytomegalovirus, human papillomavirus, Epstein–Barr virus), bacteria (*Streptococcus pyogenes*, *Neisseria gonorrhoeae*, *Treponema pallidum*, *M. pneumoniae)* or fungi (*Candida albicans*) [14,15,16,17,24,27,28,29,30,47,48,49,50].

The initial management of RIME, similar to SJS and TEN, requires prompt hospital evaluation to confirm the diagnosis, assess severity, and involve dermatology and infectious disease specialists. Supportive care is considered essential and includes skin and mucosal care, eye care, adequate hydration and nutrition, and pain management [5,42,43,51]. Patients presenting with signs of community-acquired pneumonia are recommended to receive empiric antibiotics covering *M. pneumoniae*, *Chlamydia pneumoniae*, and *Streptococcus pneumonia* (macrolide, tetracycline, or fluoroquinolones) [52,53]. Management of RIME is primarily supportive, with antimicrobial therapy directed toward the underlying infectious trigger and immunomodulatory treatment considered in selected severe cases [16,18].

This case series aims to raise awareness of RIME as a clinical complication of *M. pneumoniae* infection, highlighting diagnostic challenges, therapeutic strategies, and implications for patient care.

## 2. Case Presentation

### 2.1. Case 1

A 21-month-old male, with a medical history notable for varicella and suspected hand–foot–mouth disease two months earlier, was admitted in the Emergency Clinical Hospital for Children “Louis Turcanu” Timisoara with persistent fever, poor appetite, and painful mucosal lesions. Prior to admission, the child had developed fever up to 38.5 °C for seven consecutive days, associated with anorexia and progressive painful oral ulcerations. At admission, he appeared mildly ill, with low-grade fever (37.3 °C), marked odynophagia, and inability to feed adequately. No new medications had been administered before symptom onset, which excluded a drug-related reaction such as SJS/TEN.

**Figure 1:**
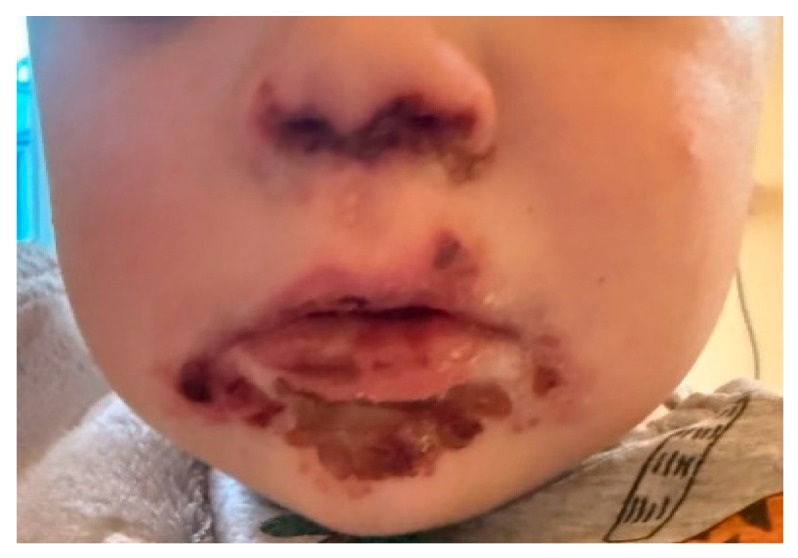
Severe ulcerative lesions of the oral mucosa with diffuse erythema and perioral honey-colored crusting, at initial presentation.

Physical examination revealed extensive ulcerations, corresponding to grade 2–3 oral mucositis, involving the tongue, buccal mucosa, hard palate, and tonsillar pillars, accompanied by diffuse mucosal erythema and edema. Honey-colored crusts were observed around the lips and perioral area (Figure 1). No other cutaneous lesions were detected.

Laboratory tests were focused on inflammatory markers, complete blood count (CBC) and immunological parametres (Table 2). Stool antigen testing was positive for adenovirus, sterile cultures from perioral crusts and mucosal lesions.

Treatment included azithromycin 10 mg/kg orally on day 1, followed by 5 mg/kg/day orally once daily on days 2–5 (maximum 500 mg/day), fluconazole 6 mg/kg/day intravenous (IV) for 4 days with prophylactic purposes, paracetamol IV, adequate hydration and targeted local mucosal care: mucosal protective agents and topical antifungal therapy—nystatin. A clear clinical improvement was noted within the first 24 h after azithromycin administration, while clinical recovery was achieved after 10 days, with complete resolution of fever and mucosal lesions, as well as normalization of oral intake.

### 2.2. Case 2

A previously healthy 12-year-old male experienced two distinct episodes of mucocutaneous disease within a six-month interval; both associated with *M. pneumoniae* infection.

The first episode occurred in October 2024 when the patient was admitted for a two-week history of persistent dry cough and rhinorrhea, followed by the acute onset of painful oral ulcerations. No new medications had been administered before symptom onset, which excluded a drug-related reaction such as SJS/TEN.

**Figure 2:**
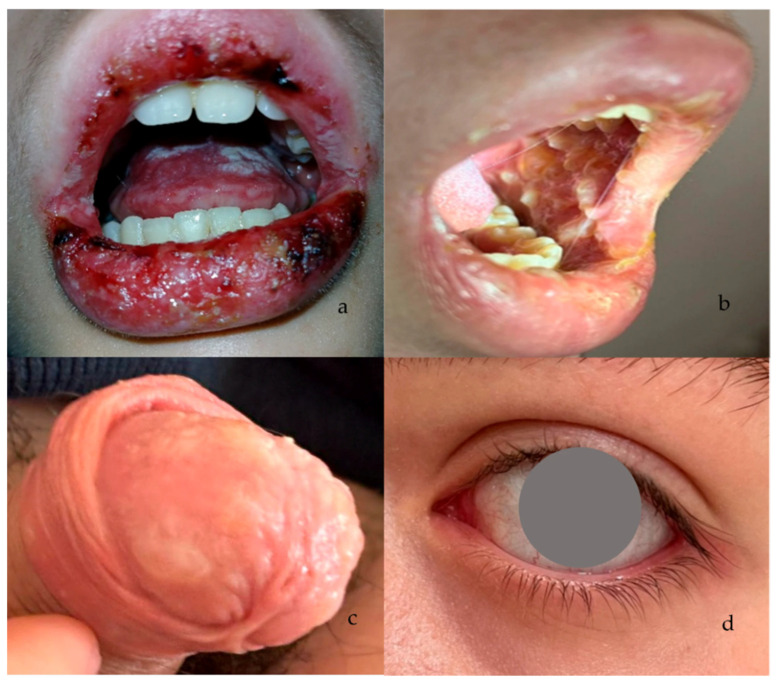
Clinical case 2: (**a**) Severe ulcerative lesions involving the oral mucosa and lips, with confluent ulcerations, hemorrhagic crusts, and marked edema, day 1. (**b**) Extensive ulcerative lesions of the oral mucosa with diffuse erythema, fibrinous exudate, and perioral honey-colored crusting, day 5. (**c**) Ulcerative lesions of the glans penis with surrounding erythema, consistent with genital mucosal involvement. (**d**) Ocular findings during the recurrence episode. Mild conjunctival injection and hyperemia indicate ocular involvement during a relapse of RIME secondary to *M. pneumoniae* infection.

At admission, the patient was afebrile but with appetite loss, presenting Grade 3 oral mucositis, characterized by confluent ulcerations, marked pain, and significant feeding intolerance. Multiple erosive and ulcerative lesions were present on the lips, oral mucosa, tongue, and pharynx, associated with hemorrhagic crusts of the lips and severe odynophagia impairing oral intake (Figure 2a). A solitary ulcer was present on the dorsal surface of the right hand, and genital mucosal involvement (penile ulcerations) (Figure 2c). No conjunctival involvement was observed, and there was no lymphadenopathy or clinical evidence of sepsis. Laboratory testing revealed modifications in inflammatory markers (Table 2). The chest X-ray was normal.

Therapy included azithromycin 10 mg/kg orally on day 1, followed by 5 mg/kg orally once daily on days 2–5 (maximum 500 mg/day), systemic corticosteroids methylprednisolone 1 mg/kg/day IV, 3 days, and prophylactic fluconazole 6 mg/kg/day orally for 4 days, together with intensive local mucosal care using antiseptic rinses, anesthetic sprays, and protective ointments.

Recovery occurred within approximately 14 days, with complete resolution of oral, genital, and cutaneous lesions.

The second episode occurred in April 2025, six months after the initial episode, when the patient was re-admitted with a recurrence of severe mucocutaneous disease, more extensive and prolonged than the first episode. The illness began with painful oral ulcerations and progressive odynophagia leading to complete feeding intolerance. No new medications had been administered prior to symptom onset, excluding a drug-induced mucocutaneous reaction such as SJS/TEN.

At clinical presentation, the patient exhibited grade 3–4 oral mucositis, characterized by confluent ulcerations, extensive mucosal erythema, marked pain, and inability to tolerate oral alimentation. Lesions involved the lips, buccal mucosa, tongue, and pharynx, with hemorrhagic crusting and diffuse mucosal swelling (Figure 2a,c). Severe odynophagia resulted in complete feeding intolerance. During hospitalization, bilateral conjunctivitis (Figure 2d) and genital mucosal involvement (ulcerations of the glans penis) developed, confirming multi-site mucosal involvement. No cutaneous vesicles or necrotic epidermal detachment were present.

Blood tests included inflammatory markers, CBC and immunological parameters (Table 2). Chest radiography was normal. Viral screening (HSV, influenza, SARS-CoV-2) and autoimmune markers were negative.

Therapeutic management included azithromycin 10 mg/kg IV on day 1, followed by 5 mg/kg IV once daily on days 2–5 (maximum 500 mg/day), empiric acyclovir 10 mg/kg IV every 8 h for 5 days, discontinued after HSV negativity), systemic corticosteroids: methylprednisolone 1 mg/kg/day IV for 3 days, tapered, fluconazole 6 mg/kg/day IV for 3 days, topical nystatin, and structured mucosal care with anesthetic and protective agents. Electrolyte correction and nutritional support were required due to impaired oral intake.

This second episode resolved more slowly, with improvement after day 5 (Figure 2b) and complete recovery by day 21, without systemic sequelae.

### 2.3. Case 3

A 12-year-10-month-old male, with no significant past medical history, was admitted with severe mucocutaneous involvement in the context of *M. pneumoniae* infection. No new medication had been administered during the six months preceding hospital admission.

Prior to admission, the illness commenced two weeks earlier with persistent dry cough and intermittent fever, initially managed by the family physician as a viral respiratory infection with symptomatic therapy. Despite transient improvement, the cough progressed and became paroxysmal and emetizing. The patient developed progressive buccopharyngeal mucositis and refusal of oral intake. After 5 days, he presented to the Emergency Department with poor general condition, severe odynophagia, and conjunctivitis.

**Figure 3:**
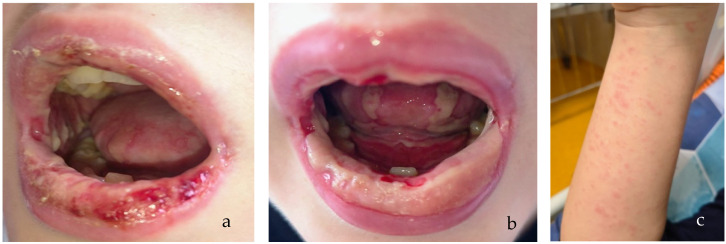
Clinical case 3: (**a**) Extensive severe oral-pharyngeal mucositis with diffuse erythema, extensive ulcerations, and fibrinous exudate involving the lips, buccal mucosa, and oropharynx, extensive fibrinous exudates—day 1. (**b**) Extensive oral mucosal ulcers in dynamic healing, early reepithelization, mild bleeding—day 7. (**c**) Maculopapular erythematous rash with scattered petechial elements on the upper trunk and extremities—day 2.

Clinical examination revealed grade 3–4 buccopharyngeal mucositis characterized by confluent ulcerations, friable mucosa, diffuse fibrinous deposits on the palate, tongue, and buccal mucosa, and marked mucosal bleeding (Figure 3a). The lips appeared carmine-red, cracked, and bleeding. Bilateral conjunctivitis and a non-pruritic erythematous macular rash with scattered petechial elements were observed on the face, trunk, and extremities (Figure 3c). Cardiorespiratory examination revealed crakles at the right lung base, while abdominal and neurologic evaluations were unremarkable. Ophthalmologic evaluation confirmed acute conjunctivitis. Dermatologic assessment confirmed a vasculitic-type rash without epidermal necrosis or blistering.

Laboratory testing focused on inflammatory markers, CBC, immunological markers (Table 2), mild coagulopathy and hyponatremia. Viral screening (SARS-CoV-2, influenza A/B) and streptococcal antigen were negative. Lung ultrasound revealed a 2 cm consolidation on the right lung, inferior lobe, accompanied by pleural reaction, consistent with pneumonia [53]. A multiplex polymerase chain reaction (PCR) respiratory panel confirmed *M. pneumoniae*. Molecular detection of *M. pneumoniae* DNA was performed using a real-time PCR platform (Rotor-Gene Q, Qiagen, Hilden, Germany), ensuring high analytical sensitivity for pathogen DNA detection using Seegen Respiratory Panel 4 (*Bordetella parapertussis*, *Bordetella pertussis*, *Chlamydophila pneumoniae*, *Haemophilus influenzae*, *Legionella pneumophila*, *M. pneumoniae*, *Streptococcus pneumoniae*) [36]. The immunologic assessment included quantitative measurement of serum immunoglobulins (IgG, IgA, IgM, IgE) and peripheral leukocyte evaluation (absolute neutrophil and lymphocyte counts) (Table 2). Results demonstrated normal IgG, IgA, and IgM levels, normal absolute neutrophil count, and no lymphopenia or T/B cell abnormalities, thereby excluding primary humoral or cellular immunodeficiency [55,56].

The evaluation specifically aimed to exclude common primary immunodeficiencies such as selective IgA deficiency, common variable immunodeficiency (CVID), or T-cell–mediated defects, none of which were supported by the laboratory findings. However, total serum Immunoglobulin E (IgE) was markedly elevated (510 IU/mL; reference <100 IU/mL), indicating non-specific immune hyperreactivity, possibly reflecting a post-infectious or atopic immune response. These findings were interpreted as consistent with an infection-related hyperinflammatory state rather than an underlying immunodeficiency [57].

Treatment included azithromycin 10 mg/kg IV on day 1, followed by 5 mg/kg IV once daily on days 2–5 (maximum 500 mg/day). The patient also received prophylactic fluconazole 6 mg/kg/day IV for 7 days to prevent progression of oral candidiasis, analgesics to reduce pain and structured supportive care (parenteral fluids, electrolyte correction, and nutritional support). Local mucosal management included antifungal and anesthetic rinses, protective gels, and emollients for the lips. Conjunctivitis was treated with a topical antibiotic–antiinflamatory solution.

The clinical evolution was gradual but favorable, and the patient was discharged after 12 days in good condition, with residual mucosal healing and fading hyperpigmentation.

## 3. Discussion

This case series illustrates the clinical spectrum of RIME. In our cases, all patients were male and originated from rural communities; however, given the small sample, no causal inferences can be drawn [5,6,20].

Our findings are consistent with published literature describing multi-mucosal involvement as the hallmark of RIME most often affecting the oral mucosa and variably involving ocular and genital sites [6,14,16,41,51,57,58]. Cutaneous involvement is frequently limited or absent, which supports the distinction from SJS/TEN in the appropriate clinical context [14,18,59]. In the present series, oral mucositis occurred in all patients; ocular involvement was observed during the recurrent episode (Case 2) and in the systemically severe presentation (Case 3), while genital involvement occurred in the relapsing case. Skin lesions were absent or limited in two cases and more extensive in the systemically severe case, similar with literature findings [4,6,14,24,25,28,29,30,48,51].

The pathogenic mechanisms underlying extrapulmonary manifestations of *M. pneumoniae* are incompletely understood and are thought to be predominantly immune-mediated rather than due to direct microbial invasion [5,15,48,59]. On the other hand, unlike SJS/TEN, RIME is infection-triggered, involves limited epidermal detachment, and shows minimal keratinocyte necrosis on histology [4,16,31,37]. In our series, the patient with the most severe systemic phenotype (Case 3) exhibited marked inflammatory activation, including elevated D-dimer levels suggestive of vascular involvement, together with markedly increased total serum IgE. Although none of the patients fulfilled criteria for primary immunodeficiency, in the systemically severe presentation (Case 3), markedly elevated total serum IgE levels were observed in the absence of quantitative IgG, IgA, or IgM deficiencies and without evidence of cellular immunodeficiency [7,12,14,48,57,59,60,61,62]. This finding was interpreted as reflecting infection-related immune hyperreactivity rather than an underlying primary immunodeficiency [59]. Total IgE testing was not uniformly available across all cases, representing a limitation of this series.

RIME is most frequently associated with previous respiratory infection, which can be documented by clinical symptoms and imaging findings, including pulmonary consolidations identified by pulmonary ultrasound examination [53].

In adolescents presenting with prominent oral and genital mucositis, particularly in the setting of recurrent episodes, Behçet disease should be considered. In Case 2, this diagnosis was excluded based on the strict temporal association with acute *M. pneumoniae* infection, absence of systemic features, and negative pathergy testing and HLA-B51, supporting classification within the RIME/MIRM spectrum [4]. Recurrent episodes have been documented, particularly in adolescents [21,23,35,37,63,64]. Our Case 2 exemplifies this relapsing phenotype, with two distinct hospitalizations occurring six months apart, both microbiologically confirmed as *M. pneumoniae*-associated. This relapsing course is consistent with previous reports and underscores that a subset of pediatric patients may experience recurrent RIME/MIRM episodes, warranting clinical awareness and follow-up [14,21,35,64].

Management in our cases was individualized according to disease severity and clinical presentation. All patients received macrolide therapy targeting *M. pneumoniae*, in addition to supportive measures including hydration, pain control, and local mucosal care. Antifungal therapy was administered when clinically indicated. In severe presentations of RIME/MIRM, systemic corticosteroids have been used in clinical practice and reported in case series, although available evidence remains limited and largely observational [4,8]. Preventive use of antibiotics is not recommended due to the risk of antimicrobial resistance [65]. Clinical outcomes were favorable in all cases. Mucosal healing occurred within 7–21 days, consistent with previous reports, and no long-term sequelae were observed. One patient exhibited transient post-inflammatory hyperpigmentation following a severe systemic presentation, which gradually resolved during follow-up [4,16,18,35,66].

The present case series provides several unique observations in the context of pediatric RIME literature. First, a relapsing phenotype was documented in an adolescent, with two hospitalizations occurring six months apart, both microbiologically linked to *M. pneumoniae*. Second, a toddler (21 months old) was included—an age group rarely represented in RIME/MIRM cohorts—thereby expanding the pediatric age spectrum. Third, one patient exhibited systemic involvement, including pneumonia and coagulation/inflammatory abnormalities, consistent with evidence that *M. pneumoniae* infections may cause marked extra pulmonary inflammation. Finally, adenovirus positivity was observed in the toddler; although adenovirus can independently trigger RIME, its coinfection with *M. pneumoniae* has been associated with more severe disease, supporting a possible cofactor role in pathogenesis [17,23,35,43].

From an epidemiological perspective, RIME remains a poorly quantified condition, with available data largely derived from case reports and small case series. Although the present report was not designed to assess prevalence, the limited number of identified cases within a single tertiary pediatric center over a defined period underscores the rarity of this entity and the challenges in capturing its true population burden.

## 4. Conclusions

This case series highlights the clinical heterogeneity of *M. pneumoniae*-associated reactive infectious mucocutaneous eruption, ranging from early-onset isolated mucositis to recurrent disease and a severe systemic inflammatory presentation. The relapsing course observed in one adolescent and the systemic involvement documented in another patient underscore the variability of disease expression within the RIME/MIRM spectrum. In all cases, outcomes were favorable under severity-adapted, supportive management.

## Figures and Tables

**Table 1:** Histopathologic and clinical differentiation between RIME, SJS, TEN, and EMM [4,5,9,16,17,18,23,31,32,37,38,39,40,41,42,43].

Feature	RIME	SJS	TEN	EMM
Etiology	Infectious—mainly *M. pneumoniae*, adenovirus, coronavirus, or Group A *Streptococcus*	Drug-induced (antibiotics, anticonvulsants, NSAIDs, Allopurinol)	Drug-induced (same as SJS)	Infectious—HSV-1/HSV-2
Prodromal phase	Respiratory symptoms (cough, fever, malaise)	Flu-like symptoms (fever, malaise)	Severe febrile prodromal	Mild prodromal; often recurrent
Mucosal involvement	More than two sites (oral, ocular, genital); frequently severe mucositis	1–3 mucosal sites; erosions common	1–3 sites; severe erosions	Usually one site (oral or ocular); mild-to-moderate
Cutaneous lesions	Sparse, non-necrotic, <10% BSA; maculopapular or atypical target-shaped	Vesicles, bullae, erosions; <10% BSA	Extensive (>30%; 10–30% = SJS/TEN overlap)	None or minimal
Epidermal detachment	Minimal or absent (<1–2% BSA)	Limited (<10%)	Extensive (>30%; 10–30% = SJS/TEN overlap)	None or minimal
Histopathology	Mild interface dermatitis with superficial perivascular lymphocytic infiltrate and focal keratinocyte necrosis; no full-thickness epidermal necrosis.	Full-thickness epidermal necrosis with sub epidermal cleavage; sparse dermal inflammation.	Identical to SJS—full-thickness epi-dermal necrosis with subepidermal separation; distinguished only by extent (>30% BSA).	Interface dermatitis with focal keratinocyte necrosis, papillary dermal edema, and lymphocytic infiltrate; no diffuse epidermal necrosis.
Trigger interval	Usually within 1–2 weeks after respiratory infection	1–3 weeks after new drug exposure	1–3 weeks after new drug exposure	1–3 days after HSV infection
Systemic findings	Mild-to-moderate; good prognosis	Possible hepatic, renal, or ocular involvement	Severe multi-organ involvement	Mild or absent systemic symptoms
Prognosis	Favorable; complete recovery	Variable; 5–10% mortality	Poor; >30% mortality	Excellent; self-limited

RIME—reactive infectious mucocutaneous eruption; SJS—Stevens-Johnson Syndrome; TEN—toxic epidermal necrolysis; EMM—major multiform erythema, NSAIDs—non-steroid anti-inflammatory drugs, HSV—herpes simplex virus; BSA—body surface area.

**Table 2:** Laboratory findings in pediatric patients with *M. pneumoniae*–associated RIME.

Characteristic	Case 1	Case 2 (1st Episode)	Case 2 (2nd Episode)	Case 3
CBC				
WBC (/mm^3^)	10.830	11.480	10.650	9.130
Neutrophils (/mm^3^)	6.460	8.270	6.360	5.120
Lymphocytes (/mm^3^)	2.790	1.780	2.760	2.510
Platelets (/mm^3^)	571 ↑	261	188	617 ↑
Inflammatory markers				
CRP (mg/L)	80.85 ↑	31.64 ↑	24.05 ↑	144.54 ↑
ESR (mm/h)	n.a.	26 ↑	12	96 ↑
Ferritin (ng/mL)	n.a.	62	n.a.	870 ↑
Immunological parameters				
IgG (g/L)	n.a.	11.86	12.05	11.78
IgA (g/L)	n.a.	2.66	2.48	1.53
IgM (g/L)	n.a.	1.57	1.20	2.36
Total IgE (IU/mL)	n.a.	34.2	n.a.	510.8 ↑
Complement C3 (g/L)	n.a.	1.65	1.67	n.a.
Complement C4 (g/L)	n.a.	0.26	0.38	n.a.
IgM *M. pneumoniae*	13	27	24	n.a.

WBC—white blood cells, CRP—C reactive protein, ESR—erythrocyte sedimentation rate, Ig—Immunoglobulin, CBC—Complete blood count. Arrow (↑)—value above the upper normal limit. CBC was performed using an automated hematology analyzer Sysmex XN-1000 (Sysmex Corporation, Kobe, Japan). CRP was measured on the Cobas Integra 400 Plus analyzer (Roche Diagnostics, Mannheim, Germany) using a turbidimetric method, results were expressed in mg/L, with values between 0 and 5 mg/L considered normal.; ferritin measured by chemiluminescent immunoassay, reported in ng/mL (reference range: 7–140 ng/mL children 1–5 years, 15–150 ng/mL children 6–12 years, 20–300 ng/mL adolescents); complement C3 and C4: measured by nephelometry, reported in g/L (C3: 0.8–1.7 g/L; C4: 0.14–0.44 g/L); total IgE measured by immunoassay, reported in IU/mL: <60 IU/mL (1–5 years), <100 IU/mL (6–9 years), <200 IU/mL (≥10 years). IgG, IgM, IgA: measured by nephelometry, reported in g/L. Age-adjusted reference ranges for immunoglobulins were: IgG 3.1–13.8 g/L (1–2 years), 4.6–16.3 g/L (3–5 years), 5.4–16.1 g/L (6–12 years), 6.5–16.0 g/L (adolescents); IgA 0.2–1.0 g/L (1–2 years), 0.3–1.9 g/L (3–5 years), 0.5–2.5 g/L (6–12 years), 0.7–4.0 g/L (adolescents); IgM 0.4–1.9 g/L (1–2 years), 0.5–2.0 g/L (3–5 years), 0.5–2.1 g/L (6–12 years), 0.6–2.3 g/L (adolescents); IgM for *M. pneumoniae* serology index (performed using a chemiluminescent immunoassay on the Euroimmun Analyzer I (Euroimmun AG, Lübeck, Germany), ISO 15189 [54]): negative (<9), borderline (9–10), and positive (>10). “n.a.” indicates parameters not available.

## Data Availability

The original contributions presented in this study are included in the article. Further inquiries can be directed to the corresponding authors.

## Abbreviations

The following abbreviations are used in this manuscript:

BSA	Body surface area
CBC	Complete blood count
CRP	C-reactive protein
CVID	Common variable immunodeficiency
DNA	Deoxyribonucleic acid
EMM	Erythema multiforme major
ESR	Erythrocyte sedimentation rate
HSV	Herpes simplex virus
IgA	Immunoglobulin A
IgE	Immunoglobulin E
IgG	Immunoglobulin G
IgM	Immunoglobulin M
IV	Intravenous
MpED	*M. pneumoniae*-related extra-pulmonary disease
MIRM	*M. pneumoniae*–induced rash and mucositis
NSAIDs	Non-steroidal anti-inflammatory drugs
PCR	Polymerase chain reaction
RIME	Reactive infectious mucocutaneous eruption
SARS-CoV-2	Severe acute respiratory syndrome coronavirus 2
SJS	Stevens–Johnson syndrome
TEN	Toxic epidermal necrolysis
WHO	World Health Organization
WBC	White Blood Cells
